# It’s a TRIM-endous view from the top: the varied roles of TRIpartite Motif proteins in brain development and disease

**DOI:** 10.3389/fnmol.2023.1287257

**Published:** 2023-12-05

**Authors:** Jane Dudley-Fraser, Katrin Rittinger

**Affiliations:** Molecular Structure of Cell Signalling Laboratory, The Francis Crick Institute, London, United Kingdom

**Keywords:** TRIM, ubiquitin, brain, neurodevelopment, neurodegeneration, glioma, infection, inflammation

## Abstract

The tripartite motif (TRIM) protein family members have been implicated in a multitude of physiologies and pathologies in different tissues. With diverse functions in cellular processes including regulation of signaling pathways, protein degradation, and transcriptional control, the impact of TRIM dysregulation can be multifaceted and complex. Here, we focus on the cellular and molecular roles of TRIMs identified in the brain in the context of a selection of pathologies including cancer and neurodegeneration. By examining each disease in parallel with described roles in brain development, we aim to highlight fundamental common mechanisms employed by TRIM proteins and identify opportunities for therapeutic intervention.

## 1 Introduction

**FIGURE 1:**
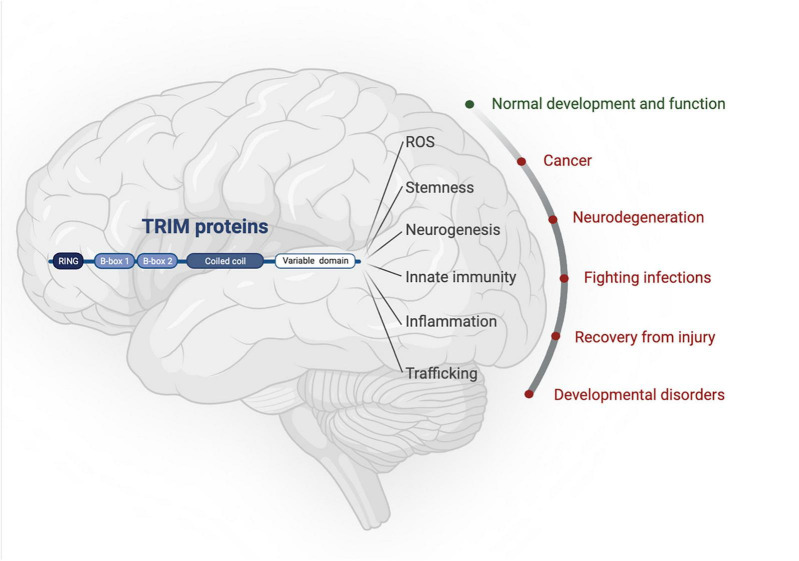
TRIM proteins have roles in normal brain development as well as being implicated in an array of neuropathologies. Created with BioRender.com.

**FIGURE 2:**
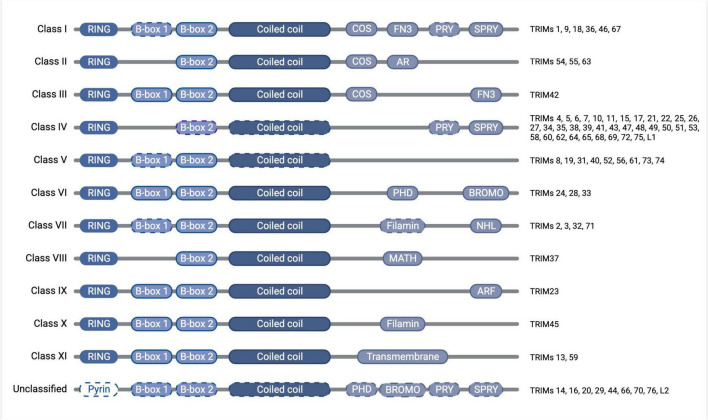
Diagram of TRIM protein domain organization and classification, with dashed outlines denoting where the domain is found only in some of the members of that class. The tripartite motif of the RING domain (E3 ubiquitin ligase catalytic domain), B-box domain(s) (functions somewhat unclear but auto-inhibition and higher-order oligomerisation have been attributed to the B-box domains of some TRIMs), and coiled-coil domain (mediates anti-parallel homodimers) is conserved throughout the family, whereas the C-terminal domains vary and confer divergent functions, which have previously been subjected to thorough phylogenetic analyses (Sardiello et al., 2008; Williams et al., 2019). Created with BioRender.com.

The tripartite motif proteins are defined by their eponymous TRIpartite Motif composed of a RING domain, one or two B-box domains, and a coiled-coil domain, which is followed by different C-terminal domains that are used to classify TRIMs into 11 classes (I-XI) (Reymond et al., 2001). The tripartite motif is highly conserved whereas the C-terminal domains vary and are proposed to offer target binding diversity, (Sardiello et al., 2008; Hatakeyama, 2017). TRIM proteins have been linked to the regulation of many cellular functions, including innate immunity, cell-cycle regulation, transcription regulation, and autophagy (Rajsbaum et al., 2014; Hatakeyama, 2017). Although TRIM protein functions have been studied across different tissues [e.g., skeletal muscle (Perera et al., 2012), the heart (Zhang J.-R. et al., 2020), and the digestive system (Chen et al., 2022)] and in multiple disease settings [e.g., immunity (Kirmaier et al., 2010; Vaysburd et al., 2013) and cancer (Hatakeyama, 2011, 2017)], here we will focus on the roles of TRIMs in brain health and disease, which themselves are diverse and extensive, with a multitude of TRIMs implicated across many different brain areas (Figures 1, 2).

Extensive research has contributed to our current understanding of the roles of each domain of the TRIM proteins. While it is now widely accepted that the coiled-coil domain is responsible for forming antiparallel TRIM homodimers, the function of the B-box domain remains somewhat unclear, though for some TRIMs they have been shown to contribute to auto-inhibition and higher-order oligomerisation (Li and Sodroski, 2008; Sanchez et al., 2014; Dickson et al., 2018). The presence of a conserved RING domain, meanwhile, has led to the assumption that the majority of TRIMs function as E3 ubiquitin ligases. E3 ligases

perform the final stage in the ubiquitination cascade by facilitating the transfer of ubiquitin onto a substrate, subsequent to the sequential action of an E1 ubiquitin-activating enzyme and an E2 ubiquitin-conjugating enzyme (Komander and Rape, 2012). RING-type E3 ligases function as adaptors to bring the substrate together with the E2-ubiquitin conjugate to mediate ubiquitin transfer.

The modification of proteins with ubiquitin or ubiquitin-like proteins [UBLs, e.g., small ubiquitin-like modifier (SUMO)], can be considered as a modular signaling code that is read by specific binding proteins to bring about particular downstream functions (Dikic and Schulman, 2023). Protein ubiquitination largely occurs through reaction with the amino group of lysine (Lys) residues, although N-terminal ubiquitination and even modification of serine and threonine residues have also been described (Wang et al., 2007; Tatham et al., 2013; Mattiroli and Sixma, 2014; Bhogaraju et al., 2016). Targets are often modified with polyubiquitin chains, the architecture of which defines the cellular response, such as degradation through the 26S proteasome induced by Lys48-linked polyubiquitin chains or activation of signaling pathways and autophagy mediated by Lys63-linked chains (Deng et al., 2000; Wang et al., 2001; Hoege et al., 2002; Xu et al., 2009; Lu et al., 2015; Ordureau et al., 2015; Yau and Rape, 2016; Grumati and Dikic, 2018).

Interestingly, there are an increasing number of reports suggesting that some RING-containing TRIM proteins do not exhibit ubiquitin ligase activity in *in vitro* assays with recombinant proteins (Guimarães and Gomes, 2018; Stevens et al., 2019; Fiorentini et al., 2020; Esposito et al., 2022). Moreover, several TRIM proteins have been ascribed additional functionalities, including SUMO ligase activity, RNA or lipid binding properties, membrane repair and transcriptional regulation, or even repressing the activity of other, ligase-competent TRIMs, largely through interactions via their variable C-terminal domains (Chu and Yang, 2011; Herquel et al., 2011; Kim et al., 2012; Lassot et al., 2018; Williams et al., 2019; Esposito et al., 2022; Randolph et al., 2022; Ma et al., 2023).

## 2 Brief overview of common pathologies of the brain

Neurodegenerative diseases result in the progressive loss of neurons and cognitive decline, as well as manifesting in a spectrum of other symptoms. Many of these diseases are characterized by inflammation, reactive oxygen species (ROS), and aberrant protein aggregation (e.g., α-synuclein in Parkinson’s disease; amyloid β and tau in Alzheimer’s disease; huntingtin in Huntington’s disease), although precise causes remain elusive (Kumar et al., 2016). Ischemic stroke (i.e., oxygen deprivation) is a major cause of death but the molecular mechanisms and druggable targets are still uncertain, although inflammation, mitochondrial dysfunction, excitotoxicity, and, oxidative stress have been implicated in the resulting neuronal cell loss (Feigin et al., 2010; Ng and Lee, 2019; Shi et al., 2019; Feske, 2021). The brain is responsible for ∼20% of the body’s oxygen demand and requires oxygen levels between 1–5% for normal function as well as to facilitate proper brain development (Silver and Erecińska, 1998; Tomita et al., 2003; Vannucci and Hagberg, 2004).

Brain infections of viruses and bacteria are targeted by microglia, the brain’s resident macrophages, which have also been shown to have roles in normal brain development (Rock et al., 2004; Neumann et al., 2009; Reemst et al., 2016; Carroll et al., 2018). However, innate neuronal and glial cell immunity is also a important line of defense: not only can they generate inflammatory cytokines to trigger the recruitment of specialist immune cells, it is also becoming increasingly understood that neural circuitry exists to control inflammation (Nair and Diamond, 2015; Pavlov and Tracey, 2017). As well as the pathogenic effects of the bacterium or virus themselves, the resulting inflammation can damage neuronal survival and activity. Infections and inflammation can also have an impact on the developing brain, with high prenatal levels of inflammatory cytokines linked with neurological development and various disorders (Zengeler and Lukens, 2021).

Autoimmunity in the brain can similarly lead to complex and devastating loss of brain function, also as a result of aberrant inflammatory signaling (Harris and Hughes, 1985; Karagkouni et al., 2013; Graus et al., 2016).

Brain cancers can fall into different classifications, but over 50% are gliomas, which are subclassified from grade 1 (less malignant) to grade 4 [most malignant, also known as glioblastoma multiforme (GBM)] (Kleihues and Cavenee, 1997). There is a striking unmet clinical need for GBM, which makes up half of glioma cases, with the 5-year survival estimated to still be approximately 5% (Delgado-López and Corrales-García, 2016). Key features of GBM include overactive receptor tyrosine kinase signaling, loss of p53, and stem-like properties that underpin treatment resistance (Venkataramani et al., 2019; Virtuoso et al., 2021; Wang et al., 2021).

Neurological developmental disorders caused by genetic mutations can manifest as wide-ranging, pleiotropic affects including perturbed behavior, motor skills, or learning/intellectual abilities (Parenti et al., 2020). Understanding the molecular pathologies of these conditions can inform fundamental biology as well as clinical management options.

## 3 The impact of TRIMs on aberrant cell division and cancer in the brain

In this section we outline roles for TRIM proteins according to the ‘Hallmarks of Cancer’ with relation to gliomas and highlight how these, in fact, relate to their functions in normal brain development (Hanahan and Weinberg, 2011).

### 3.1 Sustaining proliferative signaling

The proteomic and signaling reprogramming required to drive cancer cell growth is tightly interconnected with ubiquitination, as is the stem-like state that confers more malignant properties (Hanahan and Weinberg, 2011; Batlle and Clevers, 2017; Mansour, 2018; Yang L. et al., 2020; Zou et al., 2023). Similarly, during development a rapid expansion of neural stem cells is required to populate the growing brain (Stiles and Jernigan, 2010). It is rational, therefore, that where TRIM proteins are implicated in regulating stemness and differentiation in brain development, they may have parallel roles in gliomagenesis.

TRIM3 (class VII) is the natural place to start this section, having originally been identified as BRAin Tumor (BRAT) in *Drosophila melanogaster*. Its deletion causes optic neuroblasts and ganglion progenitors to undergo a dramatic expansion without differentiation, resulting in *brat* mutant brains reaching 10 times their normal size (Gateff et al., 1993; Arama et al., 2000). Therefore, TRIM3 was defined as a tumor suppressor and, indeed, TRIM3 loss of heterozygosity is seen in approximately a quarter of human gliomas, correlating with faster tumor growth, whilst in healthy adults it is highly expressed in the cerebellum (Boulay et al., 2009; Liu et al., 2014). It also implies a pro-differentiation role for TRIM3, which is reinforced by its ability to promote neuronal plasticity via the regulation of γ-actin and motor protein myosin V (El-Husseini and Vincent, 1999; Schreiber et al., 2015). Moreover, TRIM3 is implicated in the trafficking of GABA_*A*_ receptors in order to generate post-synaptic currents in differentiated cortical neurons (Cheung et al., 2010). In this context, TRIM3 expression is regulated by p53, an interplay that has been further explored in colorectal cancer (Han et al., 2023), but it is unknown whether this is relevant to its role in brain cancers, despite the p53 pathway being dysregulated in over 80% of glioblastoma (GBM) patients (Zhang et al., 2018). TRIM3 suppresses oncogenic C-MYC expression in GBM, resulting in a lower levels of stem cell markers CD133, Nestin, and Nanog, and subsequently reducing GBM neurosphere growth and confirming a pro-differentiation function for TRIM3 (Chen et al., 2014). It would be intriguing to understand whether these effects of TRIM3 are related to target ubiquitination, as it does for another cell-cycle regulator, p21, which is bound and ubiquitinated by TRIM3, thereby repressing cell growth (Liu et al., 2014; Raheja et al., 2014).

Another class VII member, TRIM71, appears to drive a stem-like phenotype and has functional redundancy with C-MYC pathways, as they are interchangeable in the Yamanaka stem cell reprogramming cocktail, although mechanistic details remain elusive (Takahashi and Yamanaka, 2006; Worringer et al., 2014). Despite these links with C-MYC, TRIM71 does not have an established role in gliomagenesis. However, a neural progenitor-related function may be inferred by its documented importance in mouse neural tube closure, in contrast to low levels of TRIM71 expression in adult brains (Maller Schulman et al., 2008; Uhlén et al., 2015). It is also important to understand whether these effects are brought about through the mRNA-binding translation repression function of TRIM71 (Williams et al., 2019), or its ubiquitination activity, which has been shown in a cellular context but appears to be lacking *in vitro* (Chen Y. et al., 2019; Esposito et al., 2022).

TRIM32 (class VII) is highly expressed in brain tissue, as well as being linked to neuromuscular pathologies (Kudryashova et al., 2009; Kumarasinghe et al., 2021). Specifically, TRIM32 levels are elevated in cortical neurons during development, becoming increasingly expressed in the cortical layers and depleting from the ventricular zone of the embryo over time. Mechanistically, TRIM32 has also been shown to bind and ubiquitinate C-MYC, resulting in its degradation and subsequently affecting transcriptional re-programming and neuronal differentiation (Schwamborn et al., 2009). In an intriguing parallel with normal neurogenesis, in neuroblastoma-initiating cells TRIM32 binds and ubiquitinates another MYC family member, N-MYC, at spindle poles during mitosis to drive asymmetric cell division that eventually results in tumor cell death (Izumi and Kaneko, 2014). TRIM32 also participates in a complex with Let-7a miRNA, the Argonaute components of the RISC complex, and the RNA helicase DDX6 to promote neuronal differentiation (Schwamborn et al., 2009; Nicklas et al., 2015, 2019). These findings may help us understand observations that TRIM32 overexpression promotes a differentiated, less malignant phenotype. In a murine neuroblastoma model TRIM32 enhances differentiation by catalyzing the addition of stabilizing ubiquitin chains (linkage type not defined) to the retinoic acid receptor (RARα), a factor that has well-known roles in neuronal differentiation (Sato et al., 2011; Janesick et al., 2015). Moreover, Wang et al. (2020) showed that TRIM32 also promotes the differentiation of granule neuron progenitor cells during cerebral development by inducing the degradation of SHH effector Gli1, and that loss of this regulation promotes medulloblastoma formation. This, however, can only occur once the TRIM32:PKCζ complex is disrupted, implicating this complex in stem cell maintenance (Hillje et al., 2011). In addition to development and cancer, these findings are also pertinent in limb-girdle muscular dystrophy 2H, an hereditary skeletal muscle disorder caused by TRIM32 mutations, where C-MYC regulation by TRIM32 in myogenic progenitors is implicated (Kudryashova et al., 2009; Nicklas et al., 2012).

Another TRIM that influences C-MYC is TRIM47 (class IV), whose knockdown instead reduces levels of C-MYC as well as β-Catenin and Cyclin-D1 in glioma cells. This attenuates proliferation, epithelial-to-mesenchymal transition markers, and invasive phenotypes, translating to reduced tumor burden *in vivo* (Chen et al., 2020; Ji et al., 2021). Indeed, TRIM47 expression is higher in GBM and higher grade gliomas, correlating with poorer survival rates overall, although it is also reasonably well expressed in normal brain tissue (Uhlén et al., 2015; Ji et al., 2021). The molecular mechanisms and how this relates to TRIM47 ubiquitination activity is, however, unknown.

TRIM8 (class V) expression levels also correlate with poor clinical outcomes in GBM (Micale et al., 2015). TRIM8 re-localizes from the cytoplasm of healthy neurons to the nucleus in GBM cells to establish a stem-like phenotype, with an increase in malignancy and glioblastoma stem cell markers, such as STAT3, SOX2, Nestin, and Nanog (Zhang C. et al., 2017; Venuto et al., 2019). Mechanistically, TRIM8 ubiquitinates and degrades the STAT3 inhibitor, PIAS3, to promote this pro-stem re-programming (Zhang C. et al., 2017). This is in stark contrast to the role of TRIM8 in development, where it suppresses proliferation and promotes differentiation of neural progenitor cells (Ding et al., 2021). TRIM8 knockdown thereby reduces excitatory synaptic transmission, perhaps giving context to studies showing that TRIM8 truncation mutants can result in early-onset epileptic encephalopathy, a neurodevelopmental disorder characterized by seizures and limited use of language (Sakai et al., 2016; Assoum et al., 2018). Additionally, during mouse embryonic development, TRIM8 localizes to, and therefore may regulate development of, the cerebellum, hippocampus, and cerebral cortex, which all have demonstrated roles in speech, language, and learning, and then continues to be well expressed in adult brains (Uhlén et al., 2015; Sakai et al., 2016). Better understanding of the cellular contexts and molecular mechanisms at play, including any potential ubiquitin ligase activity, may help align these seemingly opposing pro-GBM stemness and anti-neuronal stemness roles for TRIM8 and so inform glioma treatments.

TRIM11 (class IV) also has conflicting roles in neural and gliomagenic stem cells. During mouse embryogenesis, TRIM11 interacts with the neural stem cell regulator PAX6 to effect its proteasomal degradation, presumably via ubiquitination, thereby ablating PAX6-mediated regulation of a suite of neuronal effectors, as well as the expression of TRIM11 itself in an autoregulatory feedback loop (Tuoc and Stoykova, 2008; Sansom et al., 2009). Furthermore, TRIM11 knockdown mice exhibit aggregates of insoluble PAX6 and apoptosis in the cortex. TRIM11 levels, contrastingly, positively correlate with CD133^+^ and Nestin^+^ neural stem cell marker staining in GBM cells (Di et al., 2013). TRIM11 knockdown inhibits malignant GBM phenotypes *in vitro*, correlating with reduced EGFR/MAPK signaling pathway activity, although the relevance of TRIM11 ubiquitin ligase function in this context remains to be explored. Correspondingly, mouse xenograft models with TRIM11 overexpression exhibit stem-like phenotypes and enhanced tumor growth, and, importantly, clinical data shows that TRIM11 expression levels are moderate in normal brains but correlate positively with tumor grade and worse patient prognosis (Di et al., 2013; Uhlén et al., 2015).

TRIM28 (class VI), meanwhile, can function in PAX6-mediated gene expression at sites of H2K9me3 enrichment by forming a complex with it and Pauper long non-coding RNA (lncRNA), with impacts on neural stem cell function and proliferation (Pavlaki et al., 2018). A role for ubiquitin in this process has not been described. Others have shown an alternative role for TRIM28 in establishing H3K9me3 sites, which then repress endogenous retroviruses and transposable elements in neural progenitor cells to maintain stemness, and that without TRIM28 mouse embryos are not viable (Cammas et al., 2000; Fasching et al., 2015; Brattås et al., 2017; Miles et al., 2017; Grassi et al., 2019). Moreover, a co-repressor complex of TRIM28/HATS/DNMT can promote H3K27me3 marks and methylation of the promoter of SIX3, a differentiation-inducing transcription factor, which thereby reduces its expression and promotes a stem-like phenotype (Yu et al., 2020). This is, therefore, in agreement with other studies that implicate TRIM28 in glioma stemness and increased tumor grade (Jovčevska et al., 2017; Peng et al., 2019; Porčnik et al., 2021). TRIM28 can also promote growth of a variety of other cancers and drive resistance to treatments like temozolomide, although recent studies have shown this can be offset by combination treatments with DNA damage response effector inhibitors (e.g., PARP or ATM kinase) (Golding et al., 2012; Gupta et al., 2016; Czerwińska et al., 2017; Yu et al., 2020). In addition to these reports of transcription-based functions, other studies suggest TRIM28 can act as a MAGE protein-dependent ubiquitin ligase or a SUMO E3 ligase, highlighting the need for better context-dependent understanding of this protein (Ivanov et al., 2007; Doyle et al., 2010; Pineda et al., 2015; Stevens et al., 2019).

TRIM33 (class VI) is implicated in neural stem cell and glioma regulation via the TGFβ/SMAD4 and β-Catenin signaling pathways, respectively. In both cases, TRIM33 represses proliferation: murine cortical neural stem cells undergo excessive proliferation and fail to differentiate properly when TRIM33 is knocked out alongside SMAD4, indicative of potential redundancy in the pathway; whereas in human GBM, β-Catenin phosphorylation by PKCδ triggers its ubiquitination by TRIM33 and subsequent degradation, leading to suppression of tumor cell proliferation (Falk et al., 2014; Xue et al., 2015). This is supported by the observation that TRIM33 expression is lower in glioma tissue than normal brain tissue (Uhlén et al., 2015; Xue et al., 2015). However, it would be pertinent to identify the additional factors that can align the described ubiquitination of β-Catenin with the lack of detectable TRIM33 ubiquitin ligase activity *in vitro* (Stevens et al., 2019).

### 3.2 Resisting cell death

Recent studies have shown that TRIM17 (class IV) regulates neuronal cell survival or death decisions. TRIM17 expression is highest in the brain, specifically in the basal ganglia, cerebellum, and cortex (Basu-Shrivastava et al., 2021). Conversely, TRIM17 levels are lower in high grade tumors (Xiao et al., 2022). *In vitro*, TRIM17 overexpression ablates glioma cell line colony formation, aligning with data showing that TRIM17 overexpression in cerebellar neurons induces apoptosis, dependent on its RING domain (Lassot et al., 2010; Xiao et al., 2022). TRIM17-mediated neuronal apoptosis in that context is part of an orchestrated programme required for proper cerebellar developmental morphogenesis and is responsive to neurotrophic factor signaling through the PI3K/Akt/GSK signaling axis, which is, interestingly, also upregulated in glioma (Lassot et al., 2010; Yamaguchi and Miura, 2015). Understanding and harnessing the pro-apoptotic function of TRIM17 may be a powerful tool to fight glioma.

### 3.3 Evading growth repressors

TRIM45 (class X) is highly expressed in human adult brains, whilst in normal development it has been seen to be required for proper formation of the hypothalamus, hindbrain, and retina in a zebrafish model, via a mechanism that is yet to be uncovered, with ectopic overexpression resulting in aberrant expansion of these tissues (Wang et al., 2004; Choe et al., 2020). This is at odds, however, with the observation that TRIM45 expression levels are reduced in more aggressive gliomas (Zhang J. et al., 2017). On a molecular level, in glioma TRIM45 stabilizes tumor suppressor p53 by modifying it with Lys63-linked ubiquitin chains, thereby occluding its Lys48-linked ubiquitination by MDM2. Understanding how and why TRIM45 exerts seemingly both pro- and anti-proliferative effects may uncover development- or tumourigenic-dependent mechanisms.

### 3.4 Activating invasion and metastasis

TRIM67 (class I) is implicated in cytoskeletal regulation in both developmental and tumourigenic contexts in the brain. It is one of the most highly expressed TRIMs during cortex development in the late embryogenesis, particularly in neurons, where it is dispensable for proliferation but critical for post-mitotic cell functions and cortex maturation (Boyer et al., 2018, 2019; Bouron and Fauvarque, 2022). TRIM67 knockout mice have impaired spatial memory, cognition, and social functions (Boyer et al., 2018). On a molecular level, it interacts with a range of cytoskeletal, endo- and exocytotic, and synaptic regulators, with which it co-localizes at the axonal periphery and the tips of filopodia (Menon et al., 2021). In the case of the filopodial actin polymerase VASP, TRIM67 antagonizes its non-degradative ubiquitination by TRIM9 (a closely related class I family member) by competitively binding to TRIM9 (Boyer et al., 2019). The knockdown of TRIM67 in this context results in failed filopodia growth and dynamics and corresponding loss of axon turning and branching. Similarly, TRIM67 has also been shown to drive neuronal morphogenesis by regulating the SNAP47-mediated fusion of vesicles to the plasma membrane, thereby expanding the leading edge of the neuron, albeit in a ubiquitin-independent manner (Urbina et al., 2021). Remarkably, although Boyer and colleagues found that TRIM67 expression is largely restricted to neuronal cells in healthy brains, Demirdizen et al. (2022) demonstrated that it becomes aberrantly overexpressed in glial-derived oligodendrogliomas. In this context, TRIM67 promotes membrane protrusion and increased cell motility that can drive tumor growth in mouse models and correlates with alterations in Rho GTPase/ROCK2 pathway signaling (Boyer et al., 2018; Demirdizen et al., 2022). Aberrant expression of TRIM67 in non-neuronal-derived tumors in the brain is also found specifically in brain metastases from breast cancers, again correlating with regulation of invasive properties, as well as DNA damage response markers (Xuan et al., 2022). Why TRIM67 should be important particularly in non-neuronally-derived tumors, despite its neuronal functions in development, is not yet clear. Moreover, it is intriguing that a ubiquitin ligase-dependent mechanism for TRIM67 activity in the brain has not yet been identified.

TRIM37 (class VIII) is a developmentally important protein, with truncation mutations leading to MUscle-LIver-BRain-EYe (MULIBREY) nanism (i.e., individuals with unusually restricted growth). Although gross morphological brain development is normal, patients have motor and speech developmental delay, suggesting a role for TRIM37 in proper neural network formation (Karlberg et al., 2004). MULIBREY patients also experience significantly higher tumor rates and TRIM37 has also been implicated in non-MULIBREY-related cancers (Brigant et al., 2019). In glioma, for example, TRIM37 has been found to have aberrantly high expression (Tang et al., 2018). Knockdown of TRIM37 in this context correlates with reduced PI3K/AKT signaling, migration, and proliferation. If it can be understood why TRIM37 overexpression in glioma and truncation in MULIBREY can similarly lead to tumorigenesis, opportunities to treat both might be identified. The known ubiquitin ligase activity of TRIM37 has not, however, been attributed to any of these effects and might shed light on an explanation in this regard (Kallijärvi et al., 2005).

### 3.5 Inducing angiogenesis

In healthy brains, TRIM47 is developmentally regulated to facilitate hippocampal synapse development (Sharma and Banerjee, 2022). In adults, however, TRIM47 is more strongly localized to brain blood vessel endothelial cells, which may impact tumor growth by delivering oxygen and nutrients to the expanding tumor mass, thus correlating with increased TRIM47 expression in higher grade gliomas, although this has not yet been explored (Hanahan and Weinberg, 2011; Hao et al., 2019; Ji et al., 2021; Mishra et al., 2022). Moreover, it would be of interest to understand the differential molecular effects, and perhaps ubiquitination targets, that TRIM47 exerts in developing neurons versus blood vessel endothelial cells.

## 4 Regulation of protein aggregation by TRIMs

It is critical to turn on and off protein degradation during brain development to allow for the formation of different structures, such as axons, but then prevent unchecked accumulation (Saritas-Yildirim and Silva, 2014). When this is not kept under control, proteins can form pathogenic aggregates that lead to neurodegeneration (e.g., α-synuclein in Parkinson’s disease; amyloid β and tau in Alzheimer’s disease; huntingtin in Huntington’s disease) (Kumar et al., 2016). Here, we describe which TRIMs have been implicated in the aggregation of different pathogenic proteins in the brain and how this may be reflected in their roles in developmental regulation of those proteins.

### 4.1 Tau

TRIM1 and TRIM18 (also known as MID2 and MID1, both class I) are closely related proteins that can interact, localize to microtubules, and interact with cytoskeletal regulators and translation factors. Both are highly expressed in the brain during embryogenesis and are required for proper neural tube closure in *Xenopus* (Buchner et al., 1999; Suzuki et al., 2010). Moreover, an X-linked disease of midline development, Opitz G/BBB syndrome, is caused by mutations in TRIM18 that result in dysplasia of the corpus callosum and the vermis (the connection between the two lobes of the cerebellum), resulting in intellectual disabilities, as well as hypertelorism, lip-palate-laryngotracheal clefts, and some congenital heart defects (Trockenbacher et al., 2001; De Falco et al., 2003; Pinson et al., 2004; Lancioni et al., 2010). Although causative mutations (found throughout the gene with the exception of the sequence encoding the RING domain) are heterogeneous and lead to a spectrum of clinical phenotypes, dysplasia of midline structures in the brain is a central clinical feature (Pinson et al., 2004; Fontanella et al., 2008; Li et al., 2016). There have also been patients identified with TRIM1 mutations, which suggests a potential overlapping mechanism of action (Li et al., 2016). On a molecular level, mutant TRIM18 protein fails to bind to the α4 subunit of the protein phosphatase PP2A. This results in reduced TRIM18-mediated ubiquitination of PP2A, thereby increasing its activity and the subsequent hypophosphorylation of its downstream microtubule-associated substrates. One such substrate is tau, whose dephosphorylated form stabilizes microtubules. In support of this, TRIM18-deficient neurons have increased axon length and branching propensity, which then disrupt formation of the corpus callosum (Lu et al., 2013). The dysregulation of tau is also an important mechanism in Alzheimer’s (AD) and Huntington’s (HD) diseases, where it can form cytotoxic aggregates in its hyperphosphorylated form, suggesting that TRIM18-mediated degradation of tau phosphatase PP2A may contribute to neurodegeneration, although this requires further study (Schweiger et al., 2017; Rawat et al., 2022).

TRIM11 (class IV), meanwhile, has been attributed roles in the establishment of tauopathies AD and progressive supranuclear palsy (PSP, the most common cause of atypical Parkinsonism). TRIM11 is found in neurons of the cerebellum and basal ganglia in healthy adults and is also is expressed during development to regulate stem-like factors, as described in the section above on cancer, with TRIM11 knockdown resulting in the accumulation of cytotoxic insoluble aggregates of PAX6 (Tuoc and Stoykova, 2008; Jabbari et al., 2018). Similarly, in PSP, TRIM11 mutations increase levels of phosphorylated tau that can then form extensive neurofibrillary tangles (Jabbari et al., 2018; Valentino et al., 2020).

In a recent paper, TRIM11 was seen to be downregulated in the brains of AD patients and disease phenotypes in different mouse tauopathy models could be rescued by TRIM11 overexpression (Zhang et al., 2023). This was suggested to be achieved by: (a) tau SUMOylation by TRIM11, which promotes its degradation via the proteasome (although ubiquitination was not assessed here); and (b) stabilization of monomeric, non-aggregated tau through a chaperone-like function of TRIM11 via an undetermined interface. A better understanding of such molecular mechanisms of TRIMs in neurodegeneration might highlight interesting novel treatment options.

TRIM46 (class I) has been found to be key in axon specification and polarity of neurons in the cerebellum, cortex, and hippocampus (van Beuningen et al., 2015). On closer examination, TRIM46 is seen to localize proximal to the axon in parallel cross-bridged microtubules, or fascicles, a structure which is dubbed the axon initial segment (AIS) (van Beuningen et al., 2015; Fréal et al., 2019; Harterink et al., 2019; Ichinose et al., 2019; Bell et al., 2021; Bell and Zempel, 2022). Here it co-localized with Ankyrin G (ANKG) to scaffold microtubule binding proteins and recruit them to the plasma membrane, thereby facilitating cargo transport to the proximal axon (van Beuningen et al., 2015; Fréal et al., 2019). This is important as selective axonal transport is essential for neuronal polarization and function. During early neuronal differentiation, TRIM46 accumulates at the AIS via the action of KIF3/KAP3 microtubule motors, prior to the establishment of the fasciculated microtubules, requiring properly executed spatiotemporal resolution (Ichinose et al., 2019). Studies from primary neurons suggest that without TRIM46, tau is mis-sorted and improperly trafficked, whilst transformed neurons do not require TRIM46 or ANKG for axonal tau trafficking, hinting at a differentially regulated process (van Beuningen et al., 2015; Bell et al., 2021; Bell and Zempel, 2022). Given the implications of improper trafficking and accumulation of tau in neurodegenerative disease, understanding this potentially developmental distinction may prove vital. Additionally, whether TRIM46 plays simply a scaffolding platform or an active enzymatic function has not been fully explored.

### 4.2 Huntingtin, amyloid, and ataxin-1

TRIM18-induced PP2A ubiquitination increases the phosphorylation not only of tau, as described above, but also of translational inducers mammalian target of rapamycin (mTOR) and S6, thereby driving overall protein production. Moreover, the TRIM18:PP2A complex interacts with and promotes the translation of certain mRNAs, as well as interacting with several mRNA transport factors (Aranda-Orgillés et al., 2008, 2011; Liu et al., 2011; Krauß et al., 2013; Monteiro et al., 2018). This enhances the translation of pathogenic Huntingtin CAG repeat expansions in HD, as well as amyloid pre-cursor protein (APP) in AD (Müller et al., 2017; Matthes et al., 2018; Monteiro et al., 2018; Heinz et al., 2021). Indeed, elevated TRIM18 expression is observed in the temporal lobe of patients with HD (Heinz et al., 2021). Therefore, the specific depletion or inhibition of TRIM18 may be promising in helping to tackle these diseases.

Research from the Yang lab has shown that TRIM19 (also known as PML, class V) mediates the SUMOylation of poly-Q mutant Ataxin-1 and Huntingtin, thereby triggering their ubiquitination by RNF4 and subsequent clearance from cells (Guo et al., 2014; Chen et al., 2017; Zhu et al., 2020). Interestingly, TRIM11 and TRIM21 can also clear aggregates of Ataxin-1 (Zhu et al., 2020). This correlates with observations that TRIM19 can clear misfolded proteins in the nucleus, thereby preventing neurodegeneration in a polyQ expansion model of spinocerebellar ataxia (Guo et al., 2014). Given the low expression of TRIM19 detected in the brain, the extent to which this defense is employed is uncertain, unless it can be stimulated by specific triggers (Uhlén et al., 2015). In the context of cancer, meanwhile, TRIM19 has been implicated in the clearance of misfolded proteins as part of a pro-tumourigenic anti-oxidant response, in accordance with its role as an oncogenic driver as part of the TRIM19/RARα fusion protein (Chen et al., 2017). Although a developmental role for TRIM19 in the brain remains to be uncovered, SUMOylation [a suggested function of TRIM19 (Chu and Yang, 2011; Guo et al., 2014)] is extensive during brain development, particularly in the hippocampus, and TRIM19 has been implicated in driving the stem-like properties in the context of cancer (Henley et al., 2014; Zhou and Bao, 2014). Connecting these disparate lines of research and mechanisms involving TRIM19 may offer interesting answers for each disease challenge.

### 4.3 α–synuclein

TRIM41-mediated ZSCAN21 ubiquitination and degradation is inhibited by the competitive binding of TRIM17 to TRIM41 (Lassot et al., 2018). Correspondingly, increased TRIM17 levels correlate with less ZSCAN21 ubiquitination and higher ZSCAN21-induced expression of α-synuclein in PD animal models and patients (Lassot et al., 2018). Furthermore, genetic variants of TRIM17, TRIM41, and ZSCAN21 are significantly associated with familial forms of PD (Farlow et al., 2016; Lassot et al., 2018). The normal function of α-synuclein is to facilitate presynaptic homeostasis and neurotransmitter release, with perturbed α-synuclein regulation observed in autism spectrum disorders that experience synapse dysfunction (Scott and Roy, 2012; Vargas et al., 2017; Morato Torres et al., 2020). ZSCAN21 induces α-synuclein expression in primary neuronal cultures, with α-synuclein expression peaking before birth, and it would be interesting to know whether TRIM17 or TRIM41 also play a role in this context (Raghavan et al., 2004; Dermentzaki et al., 2016). Intriguingly, however, TRIM17, TRIM41, and ZSCAN21 genetic variants have also been linked to autism (Iossifov et al., 2012, 2014; Lim et al., 2017; Satterstrom et al., 2020).

Alternatively, the SUMO E3 ligase activity of TRIM11 has been seen to reduce α-synuclein fibrillar aggregates in PD and facilitate the recruitment of a SUMO-targeted ubiquitin ligases to trigger their clearance (Zhu et al., 2020). Moreover, TRIM11 overexpression can mitigate α-synuclein-mediated pathology, loss of dopaminergic neurons, and lessen PD-related behavioral phenotypes in a mouse model. Connecting all these instances is TRIM11-mediated protein degradation, which may plausibly also be attributable to its enhancement of the proteasome-activating function of USP14 that could subsequently increase overall protein turnover in the cell (Chen et al., 2018). Additional research is needed to unpick these hypotheses and align to the developmental importance of TRIM11 described in the section above.

Although TRIM21 is only expressed at low levels in the brain (Zhang et al., 2014; Uhlén et al., 2015), it is sufficient to clear both α-synuclein and tau aggregates through an antibody-mediated mechanism reminiscent of its well-documented function in clearing viral substrates, implicating it in repressing AD and PD (Mallery et al., 2010; Kondo et al., 2015; McEwan et al., 2017). It is difficult to imagine how this might be relevant during normal development, however, other than in clearing pre-natal infections (see Section 6 “The role of TRIMs in fighting viruses in the brain”).

Meanwhile, α-synuclein and tau SUMOylation by TRIM28 (class VI) results not in their degradation, but instead in their stabilization and re-localization to the nucleus, thereby increasing cytotoxicity and neurodegeneration (Rousseaux et al., 2015, 2016, 2018). The SUMOylation activity of TRIM28 has also been seen to be required for its role as transcriptional repressor, a function which is important in neurogenesis and differentiation (Lagutin et al., 2003; Ivanov et al., 2007; Yu et al., 2020). Indeed, TRIM28 is essential for post-implantation embryogenesis, including for brain development (Cammas et al., 2000; Brattås et al., 2017). Considering these lines of research together suggests that inhibition of TRIM28-mediated SUMOylation as a therapeutic strategy for neurodegeneration may impart counteracting consequences for post-mitotic neuronal fitness and function.

TRIM9 (class I) is predominantly expressed in the cerebellum, hippocampus, and cortex of adult brains, whereas during development expression is highest in the neocortex, dorsal thalamus, midbrain, basal area of the hindbrain, and spinal cord, particularly in regions of proliferation and differentiation (Berti et al., 2002). TRIM9 knockout disrupts hippocampal neuron branching, as well as brain morphogenesis more widely, thereby impairing the development of spatial learning and memory (Winkle et al., 2016; Boyer et al., 2019). In accordance with this observation, TRIM9 levels are lower in the cytoplasm of hippocampal and temporal cortex neurons of PD patients, but are enriched in intracellular Lewy body aggregates (Tanji et al., 2010). It is unclear whether this is a correlative or causative link, however, or what the molecular mechanisms are, and other studies have suggested another non-aggregation-related role for TRIM9 in PD (see Section “5 TRIMs in the regulation of cerebral inflammation”).

### 4.4 LRRK2

LRRK2 is a cytoskeleton remodelling protein that is crucial in normal neuronal morphogenesis and is one of the most frequently mutated proteins in familial PD, where it both promotes neurotoxic protein aggregation and prevents the clearance of aggregates by autophagy (Jaleel et al., 2007; Parisiadou et al., 2009). TRIM1 is therefore implicated in both neurodevelopment and PD because it can drive the ubiquitin-mediated degradation of wild-type or mutant LRRK2 (Stormo et al., 2022). Given that TRIM1 and TRIM18 are both expressed in the brain and have been shown to interact, it would be interesting to investigate whether their interplay impacts their regulation of tau and LRKK2, respectively.

### 4.5 Neurofilament

TRIM2 and TRIM3 (class VII), another pair of TRIMs with high sequence homology that can interact (Esposito et al., 2022), also have been implicated in protein aggregation-mediated neuronal pathologies. Although more studies are needed to understand the observed downregulation of TRIM3 in PD patients, which correlates with reduced PI3K/AKT pathway signaling (Dong et al., 2019, 2020), more has been uncovered regarding TRIM2. Specifically, TRIM2 can interact with and ubiquitinate cytoskeletal components, including neurofilament light chain (NF-L) (Ohkawa et al., 2001; Balastik et al., 2008; Khazaei et al., 2011). Mutations in the coiled-coil and NHL domains of TRIM2 that effect its function or stability cause Charcot-Marie-Tooth neuropathy, characterized by progressive early-onset axonal degeneration, particularly in cranial nerves, resulting in a phenotypic spectrum including muscle wasting, facial weakness, and breathing difficulties (Ylikallio et al., 2013; Magri et al., 2020). Mechanistically, TRIM2 mutations prevent the ubiquitination and degradation of NF-L, leading to neuropathic accumulations of neurofilaments in axons (Ylikallio et al., 2013). During development, however, TRIM2-mediated ubiquitination of NF-L is required for normal axonal growth, demonstrating a parallel between neurogenesis and degeneration (Khazaei et al., 2011). In our recent study, we found that TRIM2 and TRIM3 interact at lamellipodia-like membrane protrusions, reminiscent of nascent axons, and cross-regulate one another’s E3 ligase activities (Esposito et al., 2022). In light of this discovery, it will be important to interrogate the interplay of TRIM2 and TRIM3 in mediating neurodegenerative phenotypes.

Although another class VII family member, TRIM32 has also been implicated in neurofilament regulation, its knockout in fact reduces the number of neurofilaments and the diameter of myelinated motor axons, but mice present with a sarcotubular myopathy instead of neurodegeneration (Kudryashova et al., 2009). However, as TRIM32 knockout results in aberrant differentiation into excitatory glutaminergic neurons rather than inhibitory GABAergic neurons, leading to excitotoxicity and reduced overall neuronal numbers in the hippocampus and cortex, it may implicated in neurodegeneration by another means (Hillje et al., 2013; Ntim et al., 2020). Whether these effects can be connected to TRIM32 ubiquitin ligase function remains to be uncovered.

## 5 TRIMs in the regulation of cerebral inflammation

Inflammation of the brain either in adults or during development can inflict significant damage, resulting in neuronal degeneration or neurodevelopmental defects, respectively (Aktas et al., 2007; Bennet et al., 2018). Understanding how this inflammation is triggered and resolved is therefore critical.

### 5.1 NF-κB signaling and cytokine release in the brain

As discussed above, TRIM9 (class I) expression is important in brain development, particularly in promoting axonal branching, which may be relevant to axon degeneration in PD. Others have proposed an alternative role for TRIM9 in repressing PD through its inhibition of NF-κB signaling and inflammatory cytokine release, which are known to correlate with PD (Hunot et al., 1997; Kaltschmidt et al., 1997; Tansey and Goldberg, 2010). Mechanistically, TRIM9 binds β-TrCP, a component of the Skp-Cullin-F box (SCF) E3 ligase complex, which blocks SCF-mediated ubiquitination of IκBa and p100, thereby stabilizing them and hence inhibiting NF-κB (Shi et al., 2014). Interestingly, this appears to be a non-ligase-related function for TRIM9. NF-κB suppression by TRIM9 is also important during ischemic stroke, where TRIM9 upregulation in the peri-infarct area is anti-inflammatory and neuroprotective. The ability of TRIM9 to reduce NF-κB signaling may also feed into its other function in promoting axonal guidance during development, albeit in a temporally-dependent fashion, as NF-κB can be inhibitory or stimulatory in driving axonal growth, according to the developmental stage (Gutierrez and Davies, 2011). Understanding the interplay between TRIM9, NF-κB, inflammation, and axonogenesis in more detail may inform not only neuroprotective mechanisms but also treatment options in PD.

TRIM37 (class VIII) expression largely localizes to epithelial tissues in embryos, whereas in adults it is found in central and peripheral nervous systems (Kallijärvi et al., 2006). The importance of TRIM37 in regulating these systems is suggested by the motor and language developmental delays and muscle hypotonicity documented in MULIBREY patients who harbor autosomal recessive TRIM37 mutations (Karlberg et al., 2004). Interestingly, TRIM37 can ubiquitinate and degrade PPARγ, a pro-differentiation regulator of neural stem cells (Kanakasabai et al., 2012). However, this was uncovered in the context of intracerebral hemorrhage, where TRIM37-mediated PPARγ degradation in microglia promotes pro-inflammatory IL-1β release and apoptosis rather than a differentiation process (Han et al., 2019). Taken together, these data suggest that TRIM37 may act as a double-edged sword, capable of driving both development and inflammation in the brain.

Similarly to TRIM37, TRIM47 and TRIM62 (both class IV), are both upregulated and promote inflammation in the hippocampus in an ischemia/reperfusion (I/R) injury model of stroke. Their genetic ablation correspondingly reduces inflammatory signaling and caspase cleavage after I/R injury (Hao et al., 2019; Liu and Lei, 2020). I/R induces TRIM62 ubiquitination with K63-linked chains that are required for its interaction with NLRP3 (a key player in NF-κB pro-inflammatory signaling) (Liu and Lei, 2020). Unfortunately, this cannot be put into wider perspective as, to our knowledge, TRIM62 has not yet been studied in other neurological contexts. TRIM47, however, has been shown to be specifically expressed in blood vessels in the brain, the damage and rupture of which can cause stroke and also correlates with dementia (Marchesi, 2011; Vanlandewijck et al., 2018; Mishra et al., 2022). It would be interesting to understand the seeming discrepancy, however, between observed increased vessel permeability but reduced inflammatory signaling when TRIM47 is ablated, as previous studies would suggest that inflammation would lead to break down of vessel boundaries (Ono et al., 2017). Identifying the molecular mechanisms at play in these different cellular and environmental contexts may help resolve this issue.

TRIM45 (class X) has also been seen to be pro-inflammatory, with I/R triggering TRIM45-driven NF-κB signaling and cytokine production (Xia et al., 2022). This is brought about by the interaction of TRIM45 with TAB2, which it modifies with Lys63-linked poly-ubiquitin chains, promoting the formation of the TAB1/2-TAK1 complex and inducing NF-κB signaling. TRIM45 knockdown, therefore, reduces inflammation and gives more favorable outcomes after I/R. Elevated TRIM45 levels after I/R are echoed by higher expression during development so it would be interesting to understand how TRIM45 functions are determined according to circumstance (Choe et al., 2020).

Likewise, TRIM8 (class V) is pro-inflammatory after I/R- or lipopolysaccharide (LPS)-induced cerebral injury (Bai et al., 2020; Zhao et al., 2020). Upregulated TRIM8 expression after these challenges causes cerebral damage through elevated ROS or cognitive deterioration dependent on NF-κB activity, respectively. This reinforces a previous study documenting Lys63-linked ubiquitination of TAK1 by TRIM8 in response to IL-1β or TNFα stimulation, which drives subsequent NF-κB activation (Li et al., 2011). It would be intriguing to investigate whether the inflammatory responses documented in the brain also depend on this mechanism.

In the context of spinal cord injury, meanwhile, knockout of TRIM32 (class VII) results in elevated pro-inflammatory cytokine production (e.g., IL-1 and IL-10), increased cell proliferation, reduced axon initiation, and delayed recovery of motor functions (Fu et al., 2017). This phenotype finds a parallel in development, where TRIM32 reduces proliferation and promotes differentiation, largely brought about through MYC degradation and enhancing Let-7 miRNA function (Schwamborn et al., 2009). Understanding and harnessing the anti-proliferative, pro-axogenesis function of TRIM32 during development offers an opportunity to identify better treatments after spinal cord injury.

TRIM72 (class IV) has also been implicated in improving recovery from inflammatory neurological damage. By using recombinant TRIM72 protein in combination with umbilical cord-derived stem cells, it is proposed that TRIM72 can alleviate LPS-induced damage of the brain, correlating with reduced pro-inflammatory TLF4/NF-κB signaling (Guan et al., 2019b; Ma et al., 2020). TRIM72 is similarly suggested to serve a neuroprotective role after I/R injury, where it can promote survival signaling through AKT/GSK3β (Yao et al., 2016; Wu et al., 2020). In both cases, however, the specific molecular function of TRIM72 requires further study and, crucially, it is noted that TRIM72 is not expressed in the brain but rather is either exogenously delivered or possibly secreted from muscles and transported through the blood-brain barrier. This diminishes the likelihood that TRIM72 plays a role in normal brain development and function, and indeed one has not yet been described.

### 5.2 Autoimmune brain inflammation

TRIM21 (class IV) is targeted by autoantibodies in Sjögren’s syndrome, an inflammatory autoimmune condition (Tetsuka et al., 2021). Approximately 5% of Sjögren’s syndrome patients have cerebellar atrophy, with Purkinje cells predominantly affected, consistent with the observation that TRIM21 expression, whilst generally low, is enriched in Purkinje neurons of the hippocampus, cerebral cortex, and cerebellum (Zhang et al., 2014; Uhlén et al., 2015; Tetsuka et al., 2021). It remains to be understood what triggers this attack on Purkinje neurons in only a small proportion of cases.

In mice on a high-fat diet, meanwhile, brain-specific deletion of TRIM13 (class XI) potentiates insulin resistance and metabolic dysfunction, causing systematic inflammation (Qian et al., 2020). Notably, pro-inflammatory cytokine production and inflammation in the cortex, hippocampus, and hypothalamus are observed (Qian et al., 2020). This is supported by a whole-body *Trim13*^–/–^ mouse model that results in reduced type I interferon (IFN) signaling and curbs the ability of macrophages to respond to viral infection (see Section 6 “The role of TRIMs in fighting viruses in the brain”) (Narayan et al., 2014; Li et al., 2022). As TRIM13 is well expressed in the CNS and proper metabolic regulation and signaling is also critical in neuronal development, such as in neuronal polarization and axogenesis, understanding the intersection between TRIM13, metabolism, and inflammation may offer valuable insights (Williams et al., 2011; Uhlén et al., 2015).

## 6 The role of TRIMs in fighting viruses in the brain

Perhaps the most well-known role for TRIM proteins is in the innate immune response to infections (van Gent et al., 2018). In this section we outline TRIM-mediated responses to brain viral infection and draw attention to studies from other perspectives that may be interconnected.

### 6.1 HSV-1

Further to its ability to dampen inflammation in metabolic stress models, TRIM13 (class XI) also curbs NF-κB signaling during viral infection, offering a more permissive environment for replication (Li et al., 2022). This is exemplified in a mouse model of infection by the DNA virus Herpes simplex virus 1 (HSV-1), which accumulates in the brain. In *Trim13*^–/–^ mice viral load is reduced, corresponding with upregulated NF-κB signaling. Mechanistically, TRIM13 was found to add Lys6 poly-ubiquitin chains to the innate immune signaling trigger STING, which results in it being held in the endoplasmic reticulum and promotes its degradation. An alternative mechanism has also been proposed, albeit to the same effect, for TRIM13 regulation of the RNA-based encephalomyocarditis virus, which causes neurological disease (Carocci and Bakkali-Kassimi, 2012; Narayan et al., 2014). In this model, TRIM13 dampens the activity of the intracellular viral RNA sensor MDA5 to reduce type I IFN production, with the result that *Trim13*^–/–^ mice can more effectively restrict the virus. TRIM13-mediated regulation of IFNs is a compelling idea to investigate in the context of neurodevelopment and degeneration, where type I IFNs also have been seen to play a key role (Main et al., 2016; Taylor et al., 2018; Hosseini et al., 2020).

In contrast, TRIM41 represses HSV-1 replication in mouse brains by generating a signaling hub for NEMO activity (Yu et al., 2021). TRIM41, which is well-expressed in the brain, interacts with and adds Lys63-linked ubiquitin chains to BCL10, to which NEMO is then recruited and subsequently activates NF-κB and TBK1/IRF3 pathways to induce type I IFNs (Tanaka et al., 2005). This immune regulatory mechanism can be connected with three other observations in development and neurodegeneration: (a) NF-κB has an important role in axon guidance in development (Gutierrez and Davies, 2011), (b) type I IFNs have additionally been implicated in development and PD (Main et al., 2016; Taylor et al., 2018; Hosseini et al., 2020), and (c) the regulation of α-synuclein by TRIM41 is relevant for presynaptic function in both development and PD. Therefore, the regulation of α-synuclein and NF-κB by TRIM41 may have implications for neuronal function across development, neurodegeneration, and infection.

Alternatively, TRIM11 (class IV) restricts HSV-1 infection through the binding and ubiquitination of AIM2, an inflammasome component, after infection, thereby inducing the autophagic degradation of HSV-1 (Liu et al., 2016). This dampens inflammatory responses, such as the production of IL-1β and IL-18. AIM2 has been previously shown to repress dendritic branching but increase axon extension in murine hippocampal neurons during development, with an impact on spatial memory (Chen J. et al., 2019). Whether TRIM11, which is also expressed in the developing brain, can similarly ubiquitinate AIM2 in this capacity, remains to be seen (Tuoc and Stoykova, 2008). It is also interesting to note that TRIM11 both restricts HSV-1 and negatively correlates with AD pathology, given that latent HSV-1 re-activation in the brain has been suggested to increase AD risk (see Section 4.1 “Tau”) (Cairns et al., 2022).

### 6.2 Japanese encephalitis virus

TRIM21 (class IV) has a well-characterized anti-viral role (Mallery et al., 2010). Conversely, in the context of Japanese Encephalitis Virus (JEV) infection of the brain, TRIM21 appears to support viral replication as it interacts with and downregulates IRF-3 in a RING-dependent manner, thereby reducing virus-restrictive type I IFN signaling (Manocha et al., 2014). TRIM52 (class V), however, ubiquitinates and degrades JEV viral protein NS2A, possibly also supported by its ability to promote NF-κB signaling (Fan et al., 2016, 2017; Zhang P. et al., 2020). Whilst neither of these TRIMs have identifiable roles in brain development, IFN and NF-κB signaling do, as described elsewhere in this article, and their interplay with TRIM proteins remains to be fully explored in disease and developmental contexts.

### 6.3 Endogenous retroviruses

Viruses of a different kind are linked to TRIM5 and TRIM22 in the context of the brain: human endogenous retroviruses (ERVs). Multiple sclerosis (MS), which some research has suggested may have a link to ERVs, is a progressive condition that results in myelin loss in the nerves of the brain and spinal cord (Hansen et al., 2011; Nexø et al., 2011; Morris et al., 2019). TRIM5 and TRIM22, which have been shown to suppress invading viruses, have genetic variants that correlate with increased MS risk, supporting the concept of a potential viral element in MS development (Pertel et al., 2011; Di Pietro et al., 2013; Nexø et al., 2013). However, the molecular functions of TRIM5 and TRIM22 in this regard are yet to be interrogated. Alternatively, TRIM28 has been implicated in silencing ERVs during neuronal differentiation processes, and which it may be interesting to also assess in the context of MS (Fasching et al., 2015; Brattås et al., 2017).

## 7 ROS modulation by TRIMs in the brain

Although the etiology of neurodegenerative diseases, such as PD, remains unclear, reactive oxygen species (ROS) and oxidative damage have been implicated (Singh et al., 2019). Interestingly, some TRIMs have been observed to participate in this connection. TRIMs have also been shown to play a role in regulating ROS in other contexts, including ischemic or traumatic injuries and after viral insult, which may connect to their functions in development and neurodegeneration.

### 7.1 ROS in neurodegeneration

TRIM10 (class IV) appears to have a pathogenic role in PD, where its expression is increased and genetic mutations are associated with disease risk (Witoelar et al., 2017). On a molecular level, TRIM10 ubiquitinates and degrades the phosphatase DUSP6, thereby counteracting DUSP6-mediated ERK activation, ROS suppression, and apoptosis inhibition (Huang et al., 2019). TRIM3 (class VII), meanwhile, upregulates the AKT/PI3K pathway, via an unknown molecular function, thereby dampening ROS, which correlates with a reduction in PD symptoms in a mouse model (Dong et al., 2020). Interestingly, ERK, AKT, and ROS cross-talk has been demonstrated during neurite outgrowth and neuronal apoptosis (Subramaniam et al., 2003; Chang et al., 2004; Myhre et al., 2004; Zeng et al., 2010; Wang et al., 2011; Fu et al., 2020). Being able to position TRIMs in this picture may help contextualize the two contrasting outcomes of development and degeneration that are both stimulated by ERK/AKT/ROS.

As mentioned above, TRIM72 (class IV) is expressed in muscle and not the brain. However, when recombinant TRIM72 protein is administered alongside human umbilical-derived mesenchymal stem cells, oxidative stress is relieved via the activation of NRF2 and neurogenesis is promoted, thereby increasing cognitive function in a mouse model of AD (Ma et al., 2022). This is also relevant for studies of TRIM72 in regulating ROS following brain injury (see Section 7.2 “ROS in ischemic and traumatic brain and spinal injuries”).

### 7.2 ROS in ischemic and traumatic brain and spinal injuries

The mediation of NRF2 signaling by TRIM72 described above may also be linked to previous work describing a protective and regenerative role for TRIM72 in neurons damaged by H_2_O_2_, I/R, or traumatic brain injury (Yao et al., 2016; Guan et al., 2019a). In these contexts, TRIM72 reduces oxidative damage, promotes neuronal proliferation and migration, and therefore alleviates brain oedema and neurological defects. However, this is confounded by studies from heart muscle, where TRIM72 function in cellular membrane repair is hindered by oxidative stress conditions, such as elevated ROS (Cai et al., 2009; Hwang et al., 2011). By connecting research from different tissues, it may be possible to ascertain a deeper understanding of the normal and therapeutic functions of TRIM72.

TRIM32 (class VII), meanwhile, has been described to have both pro- and anti-neuronal regeneration functions. Firstly, after I/R injury, TRIM32 knockdown promotes hippocampal neuron survival through elevated NRF2 pathway activity, which protects cells against ROS-induced apoptosis (Wei et al., 2019). This is in agreement with observations that TRIM32 hinders motor function recovery after traumatic brain injury, which is attributed to increased levels of p53 superfamily member p73 and elevated apoptosis (Zhang Z.-B. et al., 2017). However, these observations are at odds with other reports of TRIM32 promoting recovery from injury. For example, TRIM32 has also been proposed to interact with ERK following spinal cord injury, resulting in improved neuronal differentiation and recovery (Xue et al., 2020). In development, TRIM32 has similarly been shown to have a role in neuronal differentiation (Sato et al., 2011; Nicklas et al., 2015, 2019; Wang et al., 2020). In order to effectively develop new therapies to treat brain injuries, particularly hypoxic damage, it would be important to disentangle how different contexts can dictate whether TRIM32 is either beneficial and detrimental to neuronal cell survival and function.

TRIM31 (class V) supports recovery after I/R by reducing ROS, driving the pentose-phosphate pathway (PPP), and maintaining mitochondrial homeostasis (Zeng et al., 2021). This is brought about by the TRIM31-mediated ubiquitination and subsequent degradation of TIGAR, a PPP inhibitor. Interestingly, neural stem cells are particularly dependent on the PPP, which is intrinsically a reducing system and, therefore, anti-ROS, and TIGAR can inhibit the PPP to drive neural differentiation (Candelario et al., 2013; Zhou et al., 2019). Although TRIM31 has been seen to promote recovery after I/R injury, its potential in regulating neuronal differentiation has not yet been explored.

## 8 Conclusion

**FIGURE 3:**
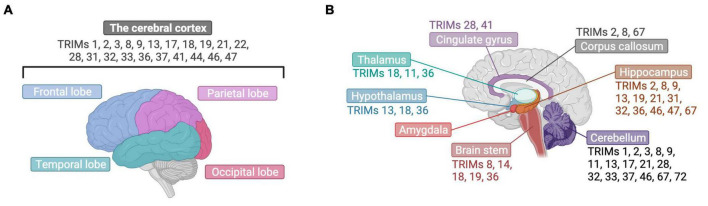
TRIM proteins have been found in varied brain structures. **(A)** The expression of many TRIMs has been detected in the cerebral cortex, presented here from a lateral view with labeling of its four different cerebral lobes. **(B)** TRIMs have been found across diverse brain structures, which are highlighted here in brain cut longitudinally and displayed laterally. Created with BioRender.com.

**FIGURE 4:**
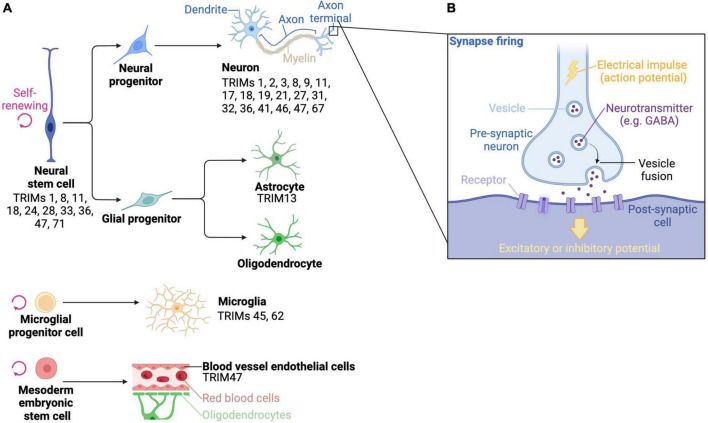
The brain is composed of complex array of cells, which express a variety of TRIM proteins. **(A)** Stylized drawings of the main differentiated cell types of the brain, as well as the stem cells that they derive from, and which TRIMs have been described in different cell types. **(B)** A diagram of a how electrical impulses are transmitted from a pre-synaptic neuron to a post-synaptic cell. Created with BioRender.com.

The roles of TRIM proteins in the brain find numerous parallels between pathological states and healthy development, with many common regulatory targets (Table 1). Intriguingly, not only are there shared functions for TRIM targets across development and disease, but there also appears to be a level of redundancy between the TRIMs, with four different TRIMs described to target MYC paralogs and eleven TRIMs capable of impacting the NF-κB pathway. Further studies are now required to understand whether this may reflect cell type- or context-specific expression patterns of these TRIMs or true functional redundancy. Some of the findings summarized above may be understood in greater depth, and any possible discrepancies resolved, by more extensive exploration and comparisons of the appropriate brain cell types and conditions in each context where TRIMs function (Figures 3, 4). Whilst the overall context-dependency of TRIM E3 ubiquitin (or SUMO) ligase activities across different bodily systems would also benefit from further scrutiny, given the multitude of physiologically-relevant putative substrates described here, the brain may prove a valuable model system to assess regulatory mechanisms. However, given the difficulty of accurately modeling this complex organ using *in vitro* models, coupled with the scarcity of human brain tissue for analysis, this is likely to pose a significant practical challenge.

**TABLE 1:** Proteins with dual roles in brain development and pathologies are regulated by TRIM family members with varying relationships to ubiquitination, structured according to the sections in the main text.

Target(s)	Cellular role of target	Relevance of target in brain development	Relevance of target in brain pathologies	TRIM(s) that ubiquitinate target	Impact of TRIM on target
**Section 3: The impact of TRIMs on aberrant cell division and cancer in the brain**					
γ-actin Myosin V	Cytoskeleton, trafficking regulation	Neuronal plasticity	Neurological disease Oncogenesis	TRIM3 (El-Husseini and Vincent, 1999; Yan et al., 2005; Schreiber et al., 2015)	Ub-induced degradation
				TRIM2 (Ohkawa et al., 2001)	Ub-induced degradation
p21	Cell cycle repression	Differentiation	Tumor suppression	TRIM3 (Liu et al., 2014; Raheja et al., 2014)	Unreported
C-MYC	Transcription regulation	Stemness	Limb-girdle muscular dystrophy 2H Oncogenesis	TRIM3 (Chen et al., 2014)	Ub-induced degradation
				TRIM32 (Schwamborn et al., 2009)	Ub-induced degradation
				TRIM47 (Chen et al., 2020)	Ub-induced stabilization
N-MYC	Transcription regulation	Stemness	Oncogenesis	TRIM32 (Izumi and Kaneko, 2014)	Ub-induced degradation
Let7a, RISC, DDX6	RNA silencing	Differentiation	Tumor suppression	TRIM32 (Schwamborn et al., 2009; Nicklas et al., 2015, 2019)	Unreported
RARα	Transcription regulation	Differentiation	Tumor suppression	TRIM32 (Sato et al., 2011)	Ub-induced stabilization
Gli1	Transcription regulation	Stemness	Oncogenesis	TRIM32 (Wang et al., 2020)	Ub-induced degradation
PIAS3	SUMO ligase, transcription regulation	Differentiation	Tumor suppression	TRIM8 (Zhang C. et al., 2017)	Ub-induced degradation
**Section 4: Regulation of protein aggregation by TRIMs**					
Pax6	Transcription regulation	Differentiation	Neurodegeneration Neurological disease Aniridia	TRIM11 (Tuoc and Stoykova, 2008)	Ub-induced degradation
VASP	Cytoskeleton regulation	Axonogenesis	Developmental defects	TRIM9 (Boyer et al., 2019)	Ub-induced stabilization
p53	Cell cycle repression	Differentiation	Tumor suppression	TRIM45 (Zhang J. et al., 2017)	Ub-induced stabilization
PP2A	Cytoskeleton regulation	Axonogenesis	Opitz G/BBB syndrome Neurodegeneration	TRIM18 (Trockenbacher et al., 2001)	Ub-induced degradation
LRRK2	Cytoskeleton regulation	Axonogenesis	Neurodegeneration	TRIM1 (Stormo et al., 2022)	Ub-induced degradation
NF-L	Cytoskeleton regulation	Axonogenesis	Neuropathy	TRIM2 (Balastik et al., 2008; Khazaei et al., 2011)	Ub-induced degradation
ZSCAN21	Transcription regulation	Synaptic transmission	Neurodegeneration Autism spectrum disorders	TRIM41 (Lassot et al., 2018)	Ub-induced degradation
α-synuclein	Vesicular trafficking	Synaptic transmission	Neurodegeneration Autism spectrum disorders	TRIM11 (Zhu et al., 2020)	SUMO/Ub-induced degradation*
				TRIM19 (Guo et al., 2014)	SUMO/Ub-induced degradation*
				TRIM21 (Mallery et al., 2010; Zhu et al., 2020)	Ub-induced degradation
USP14	Proteasome activation	Differentiation Synaptic transmission	Neurological disease	TRIM11 (Chen et al., 2018)	Ub-induced activation
Tau	Cytoskeleton regulation, trafficking	Axonogenesis	Neurodegeneration	TRIM11 (Zhang et al., 2023)	SUMO-induced degradation/disaggregase
				TRIM21 (Kondo et al., 2015; McEwan et al., 2017)	Ub-induced degradation
				TRIM28 (Rousseaux et al., 2015, 2016, 2018)	SUMO-induced stabilization
				TRIM46 (van Beuningen et al., 2015; Bell et al., 2021; Bell and Zempel, 2022)	Unreported
**Section 5: TRIMs in the regulation of cerebral inflammation**					
β-TrCP	Inflammatory signaling	Axonogenesis	Neurodegeneration Ischemic stroke	TRIM9 (Shi et al., 2014)	Blocks interaction with substrate
PPARγ	Transcriptional regulation	Differentiation	Neuroinflammation	TRIM37 (Han et al., 2019)	Ub-induced degradation
TAB2	Inflammatory signaling	Unreported	Neuroinflammation	TRIM45 (Xia et al., 2022)	Ub-induced signaling
TAK1	Inflammatory signaling	Unreported	Neuroinflammation	TRIM8 (Li et al., 2011)	Ub-induced signaling
**Section 6: The role of TRIMs in fighting viruses in the brain**					
AIM2	Inflammatory signaling	Axonogenesis	Neuroinflammation Neurodegeneration	TRIM11 (Liu et al., 2016)	Ub-induced degradation
STING	Inflammatory signaling	Unreported	Neuroinflammation	TRIM13 (Li et al., 2022)	Ub-induced trafficking
MDA5	Viral RNA sensing	Unreported	Neuroinflammation	TRIM13 (Narayan et al., 2014)	Unreported
BCL10	Inflammatory signaling/apoptosis	Neural tube closure	Neuroinflammation	TRIM41 (Yu et al., 2021)	Ub-induced signaling
IRF3	Inflammatory signaling	Unknown	Neuroinflammation Neurodegeneration Ischemic stroke	TRIM21 (Manocha et al., 2014)	Unreported
**Section 6: Reactive oxygen species (ROS) modulation by TRIMs in the brain**					
DUSP6	ROS regulation	Unknown	Neurodegeneration	TRIM10 (Huang et al., 2019)	Ub-induced degradation
TIGAR	Metabolic regulation	Differentiation	Ischemic stroke	TRIM31 (Zeng et al., 2021)	Ub-induced degradation
ERK	Pro-growth signaling	Differentiation Axonogenesis	Spinal cord injury Oncogenic Neuroinflammation	TRIM32 (Xue et al., 2020)	Unreported

*TRIM-mediated SUMOylation followed by SUMO-targeted ubiquitination by another ligase.

It is tempting to consider the extensive number of TRIMs involved in brain immunity-related processes with respect to evolution. Specifically, the adaptive immune system and the TRIM family expanded greatly during jawed vertebrate evolution, coinciding with the emergence of complex nervous systems, which may suggest a co-dependency between more sophisticated brains and the immune system, as well as potentially TRIM proteins, as has been proposed elsewhere (Meroni, 2012; Nataf, 2017; Yang W. et al., 2020; Kraus et al., 2021).

By highlighting the commonality of different players and pathways in brain development and pathologies we aimed to identify areas for future study as well as therapeutic opportunities. For example, neuronal stemness or differentiation status can be promoted or repressed by different TRIM family members, which is not just important during brain development but also during gliomagenesis, as well as being a factor in recovering from injury. Alternatively, by determining how TRIMs regulate cytoskeletal components during neurogenesis we are able to build up a more complete picture of the molecular dysfunction that leads to neurogeneration.

With regards to therapeutic development, TRIMs have been mostly studied in terms of the E3 ligase function of their RING domains to induce ubiquitin-mediated targeted protein degradation, such as in PROteolysis Targeting Chimera (PROTAC) design (D’Amico et al., 2021). However, it is notable that in many cases described above, ubiquitination is either: (a) not described; (b) does not induce target degradation; or (c) is not relevant to the effect of the TRIM (e.g., the TRIM acts an interaction scaffold) (Table 1). Therefore, rather than employing TRIMs as the ligase in a PROTAC molecule, it may be more appropriate to consider them as the target for degradation.

Moreover, given the difficulty in getting PROTACs across the blood-brain barrier due to their size and chemical properties, it may be necessary to focus on molecular glues to treat neurological pathologies (Farrell and Jarome, 2021). However, given the difficulty of fully understanding the protein-protein interface required for molecular glue prediction and design, and that the majority of molecular glues have been identified by chance, developing such strategies are likely to remain a significant challenge (Kozicka and Thomä, 2021).

Importantly, we hope that by compiling literature across different fields we are able to show that considering data from developmental and disease studies in an integrated and complimentary manner will aid the development of TRIM-based therapeutics for brain pathologies and mitigate unintended side effects (Khan et al., 2020; Békés et al., 2022; Zhang et al., 2022).

## Author contributions

JD-F: Writing—original draft, Writing—review and editing. KR: Funding acquisition, Writing—review and editing.
